# Are GP training opportunities in Northern Ireland widening or closing the gap on health inequalities? An analysis of Northern Ireland deprivation data

**DOI:** 10.3399/BJGPO.2022.0178

**Published:** 2023-06-14

**Authors:** Daniel Butler, Diarmuid O'Donovan, Jennifer Johnston, Nigel D Hart

**Affiliations:** 1 School of Medicine, Dentistry and Biomedical Sciences, Queen's University Belfast, Belfast, UK; 2 Northern Ireland Medical and Dental Training Agency, Belfast, UK; 3 Public Health Agency, Ontario, Canada

**Keywords:** health inequities, general practice, social deprivation, healthcare disparities, family practice, primary health care

## Abstract

**Background:**

Increasing the GP workforce will not necessarily level up healthcare provision. Instead, increasing GP training numbers could worsen health inequity and inequalities. This is especially true if there are fewer opportunities to learn, train, and build confidence in underserved, socioeconomically deprived areas.

**Aim:**

To investigate the representation of socioeconomic deprivation in postgraduate GP training practices in Northern Ireland (NI).

**Design & setting:**

An analysis of socioeconomic deprivation indices and scores of GP practices in NI involved in postgraudate GP training.

**Method:**

The socioeconomic deprivation indices and scores of GP postgraduate training practices were compared against general practice in NI by examining the representation of practices whose patients live in areas of blanket deprivation, higher deprivation, and higher affluence.

**Results:**

Of 319 practices in NI, 195 (61%) were registered as postgraduate training practices and had a statistically significantly lower deprivation score (3.02±0.21) compared with non-training practices (3.2±0.32), *t*(255) –2.02, *P* = 0.041. The proportion of training practices with blanket deprivation and higher levels of deprivation was underrepresented, with the current postgraduate GP training practices having more affluent populations.

**Conclusion:**

Postgraduate training practices had a statistically significant lower deprivation score and did not fully reflect the socioeconomic make-up of wider NI general practice. The results, however, are more favourable than in other areas of the UK and better than undergraduate teaching opportunities in general practice. Health inequalities will worsen if the representation of general practice training in areas of greater socioeconomic deprivation is not increased.

## How this fits in

General practice recruitment runs the risk of widening the health inequity gap if prospective trainees do not receive the opportunity to experience health care in the most deprived communities. It is known that the direction of future careers is influenced by training opportunities. Deprived areas are underserved and this study shows they remain less likely to be a training environment for future GPs. Levelling up the workforce requires, as a minimum, proportionate representation of practices in the highest need areas. However, closing the gap on health inequalities will likely require a further move towards universal proportionalism, including equitable workforce supply.

## Introduction

The need for increased general practice recruitment is well established and features frequently in headlines and political pledges, including the current UK Government party’s manifesto,^1^ as well as the policy priority to ‘level up’ society. The consequences of not ‘levelling up’ inequities in health in the UK have been exposed through the COVID-19 pandemic with *‘a clear social gradient: the more deprived the area the higher the mortality’*.^2^ While the case for increasing the workforce is well established,^3^ with ever-increasing workload and the high proportion (38%) of GPs aged >50 years,^4^ simply training more GPs will not necessarily close the health inequity and inequality gap. Rather, it potentially widens the gap, as increased GP numbers do not correspond to equal distribution across the workforce.^5^ This occurs when the training environments are not representative of the socioeconomic landscape across general practice. Boosting the numbers of GP trainees boosts the numbers of trainees within training practices, potentially underrepresenting socioeconomically deprived areas if not adequately represented as training practices. Unless increased recruitment is structured with health equity in mind, it is unlikely to help ‘levelling up’.

The ‘inverse care law’, described by Julian Tudor Hart in 1971, explained that those who had the highest need for health care are often least likely to receive it.^6^ Today, the inverse care law is more about what GPs can achieve if appropriately resourced rather than ‘good’ versus ‘bad’ healthcare quality or delivery.^7^ Not only are there fewer full-time equivalent GPs in the most socioeconomically deprived areas compared with the most affluent,^8^ but also these GPs are more stressed,^9^ manage greater rates of comorbidity in younger patients, with increased mental–physical health comorbidity,^9–13^ as well as having increased patient list sizes^11,14^ and on average shorter consultation times.^15^ To improve health outcomes for those living in the areas of highest need, there is a need for more and better primary care.

Providing postgraduate GP training opportunities is one key step to direct action when addressing the workforce issues in high-need, under-doctored areas. Fifty years ago, Tudor Hart observed that medical education propagated the inverse care law, with *'ideal medicine'* being practised under *'ideal conditions'* encouraging graduates to *'leave those who need them most and go to those who need them least'*.^6^ Previous work showed doctors' career choices were influenced by their prior experience in a specific working environment.^16,17^ Ensuring trainees are offered training opportunities in socioeconomically deprived areas is a key step to ensuring equal distribution of the future workforce,^18^ allowing early career doctors to develop an interest in general practice in different patient populations. This study examined whether postgraduate training opportunities in general practices with the highest socioeconomic deprivation are proportionately represented.

## Method

The General Medical Council (GMC) publishes the UK-approved GP practices for training in its Programme and Site Approval list.^19^ Using this data, the approved training practices across NI were identified. Deprivation indicators were collated for each general practice across NI, using the Northern Ireland Statistics and Research Agency (NISRA),^20^ where socioeconomic deprivation results are published at Super Output Area (SOA) level.^20,21^ There are 890 SOAs in NI each comprising between 1300 and 2800 people.^22^ The SOAs are ranked 1 (most deprived) to 890 (least deprived), placing 178 SOAs in each quintile of deprivation. Individual postcodes can be linked to SOAs. The postcodes of patients registered in each practice were grouped to the quintile of socioeconomic deprivation as per SOA. A deprivation profile for each general practice, using the registered patient lists living in each quintile of deprivation, was collated. To enable analysis and comparison, deprivation scores between 1 and 5 were also assigned to each GP practice in NI, as in a previous study in Scotland.^23^ If 100% of the GP patient list live in the most affluent quintile the practice score = 1, if 100% of patient list live in the most deprived quintile the score = 5. A two sample or independent *t*-test was used to measure statistical significance in the mean scores for two independent data groups,^24^ in this case training and non-training practices.

The study also compared representation of practices whose patients live in blanket deprivation, pocket deprivation, and pocket affluence. A practice with ‘blanket deprivation’ was defined as having more than half the registered patient list living in the most socioeconomically deprived 20% of NI postcodes. Practices with more than one-third of registered patients in the highest quintile for either deprivation or affluence were highlighted allowing for representational analysis of practices with higher levels of both ‘pocket’ deprivation and affluence. Quintile or 20% data cut-off points were used as this is in keeping with the NHS England Core20PLUS5 approach around health inequalities by a focus on the most deprived 20%.^25^

## Results

**Figure 1.**
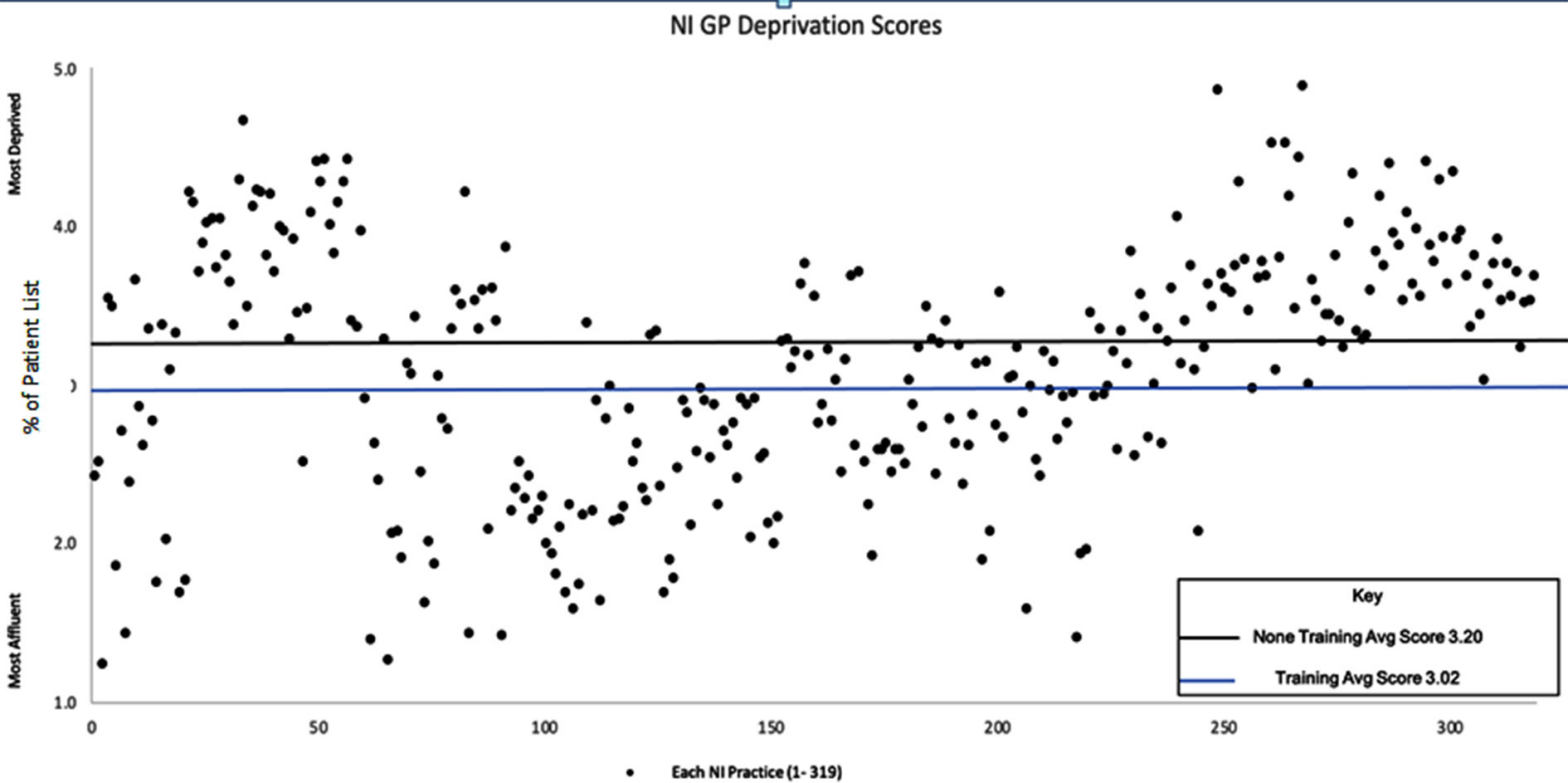
Deprivation scores for GP practices in Northern Ireland (NI). Avg = average.

In July 2022, there were 319 registered GP practices in NI. An independent *t*-test determined if there were differences in mean deprivation score between the 195 (61%) GMC-approved postgraduate training practices and 124 (39%) GP practices that did not train GPs (non-training practices). The results showed that training practices had a statistically significantly lower deprivation score (3.02±0.21) compared with non-training practices (3.2±0.32), *t*(255) –2.02, *P* = 0.041. Figure 1 shows the socioeconomic deprivation score for all NI practices.

**Figure 2.**
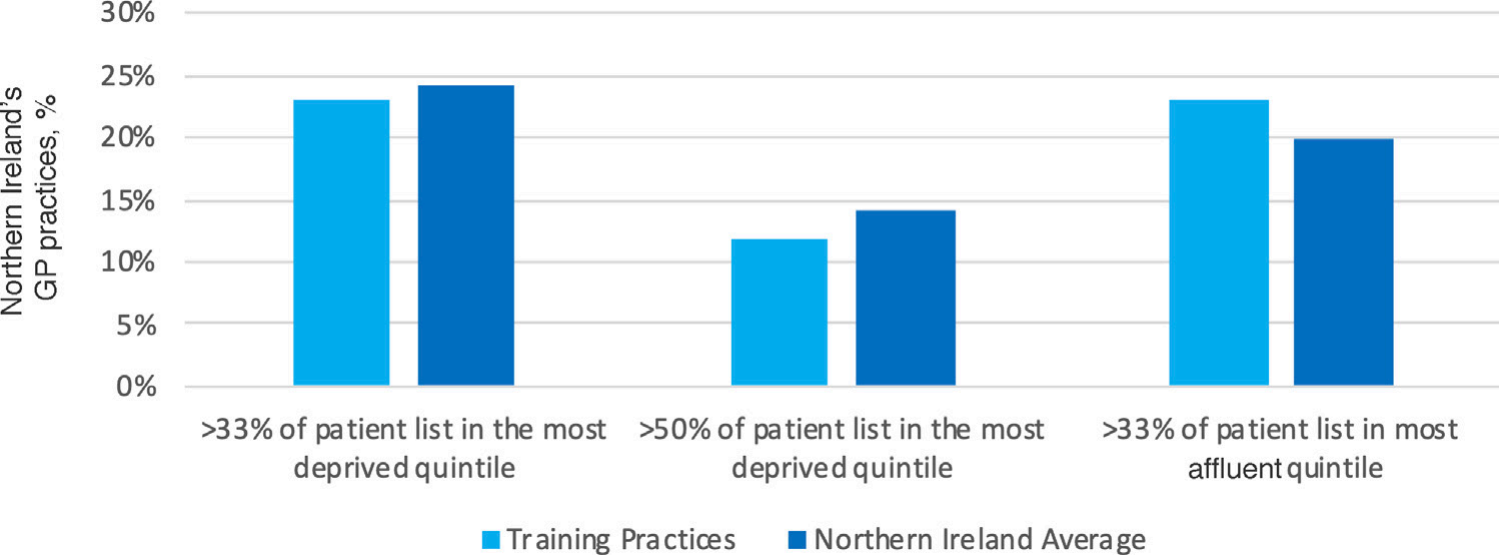
Comparing patient lists of postgraduate training practices and all of Northern Ireland’s practices

In NI, 46 practices were in areas of blanket deprivation, defined as >50% of the registered patient list living in the most socioeconomically deprived quintile. Twenty-three practices with ‘blanket deprivation’ were registered training practices, making up 12% of the training practices. Both practices with ‘blanket deprivation’ higher levels of ‘pocket deprivation’ (defined as >33% of the patient list living in the most deprived quintile) had lower representation when compared with NI as a whole (Figure 2). Conversely, more affluent practices (defined as >33% of the patient list in the most affluent quintile) were overrepresented as training practices, illustrated in Figure 2.

**Figure 3.**
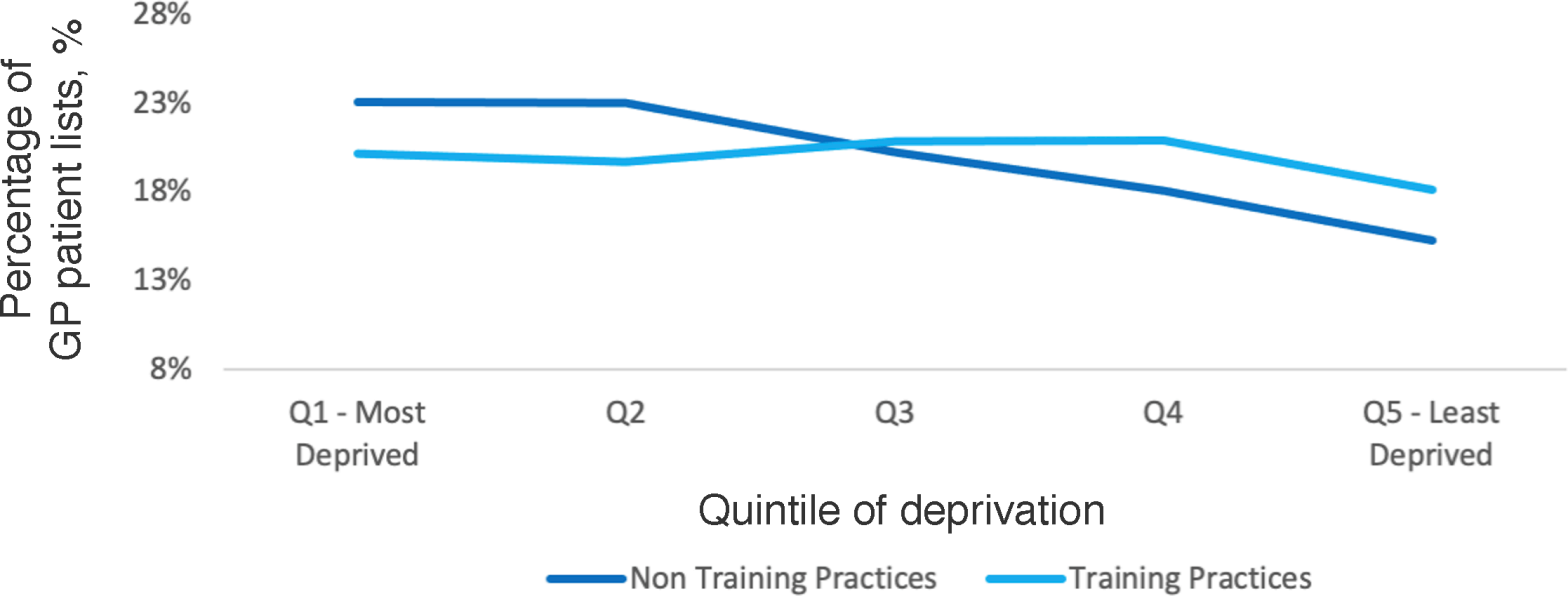
Mean percentage of patients in each quintile of deprivation

A gradient can be seen between the postcodes of registered patients in training and non-training practices; non-training practices have higher percentages of patients living in the most socioeconomically deprived quintiles, with training practice populations living in more affluent quintiles (Figure 3).

## Discussion

### Summary

It was found that the postgraduate training opportunities in areas of socioeconomic deprivation were underrepresentative of general practice in NI. Despite half of all practices with blanket deprivation being postgraduate training practices, double that seen in comparable work in Scotland and Yorkshire,^26,27^ they were still underrepresented. Practices with pocket deprivation (more than one-third of patients in the most socioeconomically deprived quintile) were more closely representative. Postgraduate training practices were more likely to be in areas of higher socioeconomic affluence, compared with practices not involved in postgraduate GP training.

### Strengths and limitations

This work is strengthened by the national coverage (whole of NI), using the externally validated NISRA deprivation indices and the GMC-approved training list, confirmed by the NI training deanery. While the data on whether these training posts were filled are not readily available, it is likely, given the increasing numbers of NI GP trainees, that most of these training practices are being utilised. The context of NI is also significant, with more than one in three people (37%) living in socioeconomic deprivation, compared with approximately one in five in England, Scotland, and Wales (18%–22%).^28^ The higher prevalence of deprivation in NI means that the involvement of practices working in areas of underserved and socioeconomically deprived populations is likely to be underestimated within the wider UK context. Consequently, postgraduate GP trainees in NI are more likely to be exposed to deprivation within the primary care setting than counterparts across the UK. An examination of whether working in a socioeconomically deprived area is a positive experience, or whether trainees are aware of the demographic of their training practices and the possible implications to clinical practice, was beyond the scope of this study.

### Comparison with existing literature

Registered practices in NI’s postgraduate training scheme are more closely representative of the general practice socioeconomic landscape when compared with socioeconomically deprived practices in Scotland and parts of England. Initially when the Scottish Deep End Project (a GP-led initiative in underserved areas with high unmet needs) was established, one-quarter of the most socioeconomically deprived 100 practices were involved in postgraduate training.^23,26^ Similarly, 27% of Yorkshire and the Humber Deep End practices were involved in training.^27^ This compares with 50% of ‘deprived’ or ‘Deep End’ practices being registered training practices in NI. Further, the recruitment of postgraduate training practices is more socioeconomically representative than undergraduate teaching practices within NI.^29^

### Implications for research and practice

This article set out to investigate whether postgraduate GP training proportionally represented practices serving the most high-need, socioeconomically deprived areas. In NI, postgraduate GP training practices have fewer patients from socioeconomically deprived areas. This raises the following question: why are GP practices in socioeconomically deprived areas less likely to be postgraduate GP training practices? As authors, it is suggested the lower number of GPs,^8,14^ higher patient lists,^10,11^ increased consultation demand, and patient complexity^13,15^ in areas of socioeconomic deprivation are likely barriers. There may also be physical barriers, such as the availability of space,^30^ especially in inner-city locations. However, more research is required to explore the barriers.

Despite the mean deprivation scores of training and non-training practices being relatively small, 0.18, it is not only statically significant but also clinically significant, as any difference, regardless of how small, potentially widens the distribution of the GP workforce and does not close the gap or level up. Given the socioeconomic context of NI, it is unlikely that NI GP trainees are only practising *'ideal medicine under ideal conditions'*, but further work is required to continue recruiting practices with higher levels of socioeconomic deprivation. Possible development within the NI GP training structure could include the creation of tailored training programmes for general practice in high-need areas, such as those seen in the North Dublin City GP Training Programme,^31^ GP Trailblazer programme,^32^ and the Deep End Pioneer Scheme.^33,34^ These areas are underdoctored and therefore intentional training and support of postgraduate training is required, moving beyond just achieving proportional representation towards 'proportionate universalism' within training opportunities. Proportionate universalism has been defined as the *'resourcing and delivering of universal services at a scale and intensity proportionate to the degree of need'.* ^35^ As the workforce in socioeconomically deprived areas is already depleted, and GP training is increasing, additional enhanced models of training that reflect proportionate universalism principles, seen in other areas of the UK and Ireland, should be considered. Not doing so may likely worsen health inequalities, as increasing GP training numbers will not correspond to equal distribution of GPs across the workforce.^5^ The results have shown GP training in NI is well-positioned to improve the distribution of the future GP workforce proportionately. With the goal of ‘levelling up’ general practice, it is a strong platform to launch a deprivation-focused training programme, moving closer to a model of proportionate universalism in the general practice workforce and closer to health equity for all.

## Data

The dataset relied on in this article is available from the corresponding author on reasonable request.
